# Editorial: A new era in the treatment of food allergy

**DOI:** 10.3389/fimmu.2026.1785706

**Published:** 2026-03-23

**Authors:** Dale T. Umetsu

**Affiliations:** Pediatrics, University of California, San Francisco, San Francisco, CA, United States

**Keywords:** allergen immunotherapy (AIT), allergy, anti-IgE, food allergy, immunotherapy, omalizumab, tolerance, Xolair

In February 2024, the United States FDA approved a new therapy, omalizumab (Xolair), for the treatment of food allergy, launching a promising new era. Omalizumab is a biologic medication, specifically a monoclonal antibody (mAb) against IgE, designed to neutralize IgE, the antibody that causes allergies and food allergy. In this breakthrough authorization, the FDA approved omalizumab for the treatment of individuals 1 year and older with any IgE-mediated food allergy. As such, omalizumab is the first FDA approved treatment for any food allergy beyond peanut, the first treatment for any food allergy in adults, the first treatment for patients with multiple food allergies, the first treatment for any child 1-4 years of age, and the first mAb approved for the treatment of food allergy.

These “firsts”, make omalizumab a game changer in the treatment and management of food allergy. Why? Because, as a biologic, omalizumab is superbly effective in limiting allergic reactions associated with food exposures in patients. It does this by limiting the ability of mast cells and basophils to drive allergic reactions, in a manner that is orders of magnitude greater than any prior medication (e.g., anti-histamines). In addition, because omalizumab has been used in patients including children for more that 20 years (first approved in 2003 for the treatment of allergic asthma, and later approved for pediatric asthma, chronic idiopathic urticaria and nasal polyps), it has a well-known safety track record, so that it can confidently be used in patients without fear of serious side effects. To be fair, it does have a black box warning for anaphylaxis with omalizumab is rare, and precautions are routinely instituted to minimize this problem. In addition, it is a mAb, and therefore requires subcutaneous injections, which can be problematic especially in children, who commonly have food allergy. However, it is now formulated as an autoinjector (for children 12 years and older), which simplifies and eases administration to patients. The availability of such an effective medication changes the whole paradigm for the treatment and management food allergy, generating great excitement in the food allergy community. Finally, the success of omalizumab for food allergy has also attracted great interest in the broader pharma industry, which now is captivated to develop additional pharmacotherapies for food allergy, which are likely to benefit patients in the future.

It should be noted that the only other FDA approved medication currently available for food allergy, Palforzia, which was approved in 2020 for peanut allergy in children 4-18 years of age, will be voluntarily discontinued in mid 2026, according to its manufacturer, Stallergenes Greer. This move, unrelated to the safety, quality or efficacy of Palforzia, will leave omalizumab as the only FDA approved medication for food allergy.

Nevertheless, the availability now of omalizumab for food allergy initiates a new era in food allergy, in which the treatment paradigm for food allergy will change, and is already changing. The new possibilities enabled with omalizumab are discussed in this Research Topic of Frontiers in Immunology, which I have been given the privilege of co-editing. We have chosen, and are excited to present, seven articles for this Research Topic that discuss several topics:

- Changes in treatment and management paradigms for food allergy that come with the availability of omalizumab. Omalizumab has been clinically available to food allergy patients for just a year, and so it is still novel, with clinicians still exploring the best ways to use it. For example, which patients will benefit most from it (e.g., patients with multiple food allergies or those with a history of severe or frequent reactions), or when to use it (e.g., intermittently before foreign travel or when students leave home for college, where eating at restaurants or cafeterias is required with reduced control of food allergen content of meals, and when risk taking behaviors may be problematic). Or, when should treatment be stopped, and whether omalizumab should be used in combination with oral immunotherapy (OIT), since omalizumab can greatly facilitate OIT, either with single food OIT or with multiple food OIT. Other questions include how to identify the few omalizumab non-responders, and how careful should patients be in avoiding problematic foods when on treatment with omalizumab?
- Cost-effectiveness analysis, which could determine how available omalizumab may be in current health care practices, i.e., whether health care payers will approve the therapy for broad use.
- Also discussed are new therapies in the pipeline for food allergy, their mechanisms of action, and the safety and effectiveness of these new therapies.
- The articles also discuss how new therapies for food allergy are changing treatment paradigms and clinical practice, and extending patient expectations, from bite safe remedies to perhaps focusing on potential cures that allow adding the problematic food to the diet.

The seven specific articles in this Research Topic are listed here:

1. Scientific Developments in Understanding Food Allergy Prevention, Diagnosis, and Treatment of food allergy, by Hund et al.

In this article, the authors provide a useful and up to date overview of food allergy, the epidemiology and immunological mechanisms of food allergy, as well as the known risks for developing food allergy, the available prevention strategies, diagnostic strategies, current treatments, and some possible future therapies. The excitement in the field of food allergy is not just about the new treatments, but the broad nature of new developments for many aspects of food allergy.

2. Anti-IgE therapy versus allergen-specific immunotherapy for food allergy: weighing the pros and cons, by Kulis et al.

The authors discuss the advantages and disadvantages of using omalizumab versus allergen immunotherapies (AITs), and the potential benefit of combining both approaches in young children. Given that food allergy patients are extremely heterogeneous, choosing the best therapeutic approach for any given patient requires understanding of the specific benefits and side effects of each, and which patient may benefit most with each. Importantly, because each approach is still relatively new, the paradigms for treatment are evolving as we speak.

The authors discuss the fact that omalizumab is effective for any food, suggesting that omalizumab might be best for patients with multi-food allergy. It may also be useful in patients with greater disease severity, those with concomitant asthma and environmental allergies and who are not averse to injections. They also discuss the three forms of allergen immunotherapy (AIT): oral (OIT), sublingual (SLIT) and epicutaneous (EPIT). AIT has disease modifying effects, in contrast to omalizumab, which does not and where efficacy ends within weeks after efficacy ends shortly after the injections stop. With AIT, long term remission is possible in some patients, particularly in very young children and those with relatively low baseline food-specific IgE levels. OIT though, (in particular with Palforzia for peanut allergy) is associated with very frequent allergic reactions. The authors also mention that combining OIT (for any food) with omalizumab can enhance the safety and speed the up-dosing stage of OIT. This combination is a promising approach, particularly since omalizumab injections might be stopped after desensitization or improvement of tolerance to the food is achieved, eliminating the need for potentially life long therapy with omalizumab. However, additional studies are needed to understand the optimal schedule for dual treatment.

3. Considerations for biologics as front-line treatment in allergic diseases, by Kothari et al.

In this article, the authors discuss the role of omalizumab in the management of IgE-mediated food allergy, weighed against other approaches used for food allergy. They discuss whether we should consider omalizumab as a first-line treatment, particularly since it is so effective in increasing the amount of food that is tolerated. The discussion also addresses health economics and patient preferences. In addition, the authors discuss the benefits of oral immunotherapy (OIT), either alone or in combination with omalizumab, which can enable faster and safer OIT up-dosing.

4. Emerging Diagnostic and Therapeutic opportunities in food allergy, by Balsiger et al.

The authors review our current understanding of how the breakdown of oral tolerance leads to food allergy. In addition, they review current and new diagnostic methods, including basophil activation (BAT) and mast cell activations (MAT) tests and oral food challenge. A significant part of this review discusses food allergen immunotherapy, including OIT, SLIT and EPIT. In addition, the authors discuss emerging immunization strategies, including the use of recombinant wildtype allergens, hypoallergens as well as carrier-bound allergens or allergen peptides in conjunction with different adjuvants. Omalizumab’s mechanism of action is discussed, which may include not only neutralizing free IgE but also actively removing IgE from its receptor, FcεR1. In addition, the authors comment on new therapeutic options in clinical trials or under development, including next generation of anti-IgE molecules, with improved efficacy of removing IgE from FcεR1; passive immunization approaches with allergen-specific IgG; and the use of small molecule inhibitors targeting JAK1 or BTK. Finally, the authors discuss the advantages of combining omalizumab with AIT.

5. Epithelial immunotherapy for food allergy in children: A systematic review and meta-analysis, by Chen et al.

This article reviews the published randomized controlled trials of EPIT performed by DBV, the company pursing the commercialization of a device that dispenses small amounts of allergen to the skin. It is an excellent summary of the benefits and safety of EPIT, a therapy that will soon likely be approved by the FDA for young children with peanut allergy.

6. Role of human antigen-specific T cells in tolerance and IgE-mediated allergic reactions to food, by Lopez-Fandino.

While the role of regulatory T cells (Tregs) is well described and accepted in murine and human immunity, their role in human food allergy is controversial, in part because they are difficult to detect, due to their small numbers and heterogeneity in food allergic patients. The author reviews many of the studies describing inhibitory Tregs in food allergy, natural tolerance or desensitization, and suggests that more definitive evidence supporting a role for allergen-specific Tregs in food allergy is still needed. She also describes other T cells involved in food allergy, including pathogenic Th2 cells, and reviews our understanding of mechanisms that underlie food allergy and its resolution.

7. Gut Commensal Bacteria-derived polysaccharide sub-micron particles induce antigen-specific, tolerogenic responses, by Harriman et al.

Gut commensal bacteria are now thought to play an important role in protecting against the development of food allergy. While administration of live bacteria is being investigated as a therapeutic for food allergy, it is possible that bacterial components may also play a role in reversing food allergy. Importantly, polysaccharide A (PSA), a component of *Bacteroides fragilis*, a known beneficial species, has been studied intensely in the past, primarily in mouse models, for its ability to enhance oral tolerance. In this article, the authors provide evidence that orally administered PSA sub-micron particles loaded with allergen, may serve as a therapeutic model for inducing tolerance in food allergy.

